# The Impact of Autistic Traits on Self-Recognition of Body Movements

**DOI:** 10.3389/fpsyg.2018.02687

**Published:** 2019-01-10

**Authors:** Joseph M. Burling, Akila Kadambi, Tabitha Safari, Hongjing Lu

**Affiliations:** ^1^Department of Psychology, University of California, Los Angeles, Los Angeles, CA, United States; ^2^Department of Statistics, University of California, Los Angeles, Los Angeles, CA, United States

**Keywords:** autism-spectrum quotient, self-recognition, body movements, biological motion, visual perception

## Abstract

Despite the sparse visual information and paucity of self-identifying cues provided by point-light stimuli, as well as a dearth of experience in seeing our own-body movements, people can identify themselves solely based on the kinematics of body movements. The present study found converging evidence of this remarkable ability using a broad range of actions with whole-body movements. In addition, we found that individuals with a high degree of autistic traits showed worse performance in identifying own-body movements, particularly for simple actions. A Bayesian analysis showed that action complexity modulates the relationship between autistic traits and self-recognition performance. These findings reveal the impact of autistic traits on the ability to represent and recognize own-body movements.

## Introduction

The concept of the “self” has widely been considered to play a crucial role in supporting the human ability to communicate with others (Anderson, 1984). Ornitz and Ritvo (1968) suggested that children with autism spectrum disorder (ASD) may suffer from a basic impairment in self-awareness, which interferes with the development of social interaction and communication with others. To test this hypothesis, a number of experimental studies have investigated whether individuals with ASD exhibit impairments in visual self-recognition. The prototypical task used to assess visual self-recognition ability in children with ASD is the mirror self-recognition procedure—examining children's responses to their reflections in mirrors. Specifically, a child views herself in a mirror after a small amount of rouge has been secretly applied to his or her nose (Neuman and Hill, 1978; Dawson and McKissick, 1984; Spiker and Ricks, 1984). These studies have largely found that most children with ASD behaved similarly to children in a control group: they touched the nose, or verbally referred to the rouge on the nose, demonstrating recognition of their own faces in the mirror. Hence, based on evidence from the mirror-mark test, it appears that self-recognition may be intact in ASD (although a developmental delay in autism has been reported, e.g., Ferrari and Matthews, 1983).

However, other researchers have pointed out that simply passing the mirror-mark test is not sufficient to establish possession of self-identification ability. For example, many infants who pass the mirror-mark test also attempt to wipe a non-existent mark off their own face when they observe that other people have a mark on their face (Lewis and Brooks-Gunn, 1979; Johnson, 1983; Mitchell, 1993). These findings raise a possibility that the mirror-mark test may not probe the core perceptual representation underlying self-recognition, and hence is not sensitive enough to identify the impact of ASD on self-recognition from visual input.

Another concern is that stimuli used in previous studies of self-recognition may not solely assess self-representation. For example, we have rich experiences from daily life of our own faces and own speech, as we often see our faces in mirrors or photos and listen to our own voices in conversations. To investigate the perceptual representation of the self, we need stimuli that can decouple high familiarity with perceptual experiences from self-recognition ability. A good candidate to investigate involves own-body actions, an extensive stimulus set that we rarely witness in our daily life. Accordingly, tasks requiring identification of the self through own-body movements can shed light on the core of self-recognition ability, without relying on visual experience. In addition, the recognition of human body movements requires an especially tight coupling between perception and action, in that these processes share a common representational platform (Prinz, 1997; Casile and Giese, 2006). Studying self-recognition of own-body movements provides a unique window to examine the interplay between perception, action, and self-recognition ability.

Several studies have reported that humans are able to identify their own-body movements, even when the actions are reduced to a dozen discrete dots representing joint movements in point-light displays (Loula et al., 2005). In fact, previous research suggests that people are even more accurate in identifying the self from own-body movements than in recognizing familiar friends from their actions (Cutting and Kozlowski, 1977; Loula et al., 2005). In addition, visual representations of own-body movements have been found to be object-centered. Jokisch et al. (2006) used point-light displays portraying walking actions to demonstrate that self-recognition of own walking actions is viewpoint-invariant, whereas the recognition of a familiar friend from walking point-light displays is viewpoint-dependent, favoring the frontal view (Jokisch et al., 2006). This characteristic reveals a fundamental difference between the action representation of the self and of others.

The ability to extract the “self” and infer “identity” from impoverished point-light displays highlights the remarkable capacity of the human visual system for biological motion perception—the ability to construe rich social information through the kinematics of body movements (Adolphs, 2003; Thurman and Lu, 2014). Several studies have investigated biological motion perception in autism. Individuals with ASD show poorer performance in extracting emotional content from body movements (Moore et al., 1997; Hubert et al., 2007; Parron et al., 2008; Nackaerts et al., 2012), reduced adaptation to action categorization (van Boxtel et al., 2016), and impairments in some action detection and discrimination tasks (e.g., Blake et al., 2003; Koldewyn et al., 2009; Annaz et al., 2010; Nackaerts et al., 2012).

Behavioral differences in the processing of biological motion information have not only been reported when comparing ASD and control groups, but also in typical populations with varying degrees of autistic traits. The Autism-Spectrum Quotient (AQ) questionnaire is the most common measure of self-reported autistic traits (Baron-Cohen et al., 2001). Recent evidence has identified an overlapping genetic and biological etiology underlying ASD and autistic traits (Bralten et al., 2017), in addition to behavioral overlap (Baron-Cohen et al., 2001; Robertson and Simmons, 2013). Several studies of biological motion perception have reported an association between AQ scores and performance on various tasks. For example, people with a higher number of autistic traits show impairments in interpreting social actions (Bailey et al., 1995; Kaiser et al., 2010; Ahmed and Vander Wyk, 2013; van Boxtel et al., 2017). Individuals with more autistic traits showed poorer performance in judging the facing direction of a walker, a task requiring contextual integration (Miller and Saygin, 2013). In addition, people with more autistic traits showed less perceptual adaptation to actions (van Boxtel et al., 2016) and less neural adaptation in the right posterior superior temporal sulcus, the key brain region for perceiving, and understanding human actions (Thurman et al., 2016).

However, previous work has only examined action recognition or categorization and compared such ability between people with different degrees of autistic traits. Therefore, it remains unknown whether the ability to identify own-body movements is systematically impacted by the degree of autistic traits. If people with a high degree of autistic traits lack a clear perceptual representation of their own-body movements, we hypothesize that they may exhibit worse performance in self-identification than people with a low degree of autistic traits.

In the present study, we recorded body movements of individual participants and conducted a self-recognition task after a considerable delay (~2.75 months). We then examined the relation between autistic traits and self-recognition performance, and further examined whether this relation was modulated by action complexity (as more complex actions involve increased motor planning and distinctive movement styles for different individuals).

## Methods

### Participants

Forty-three undergraduate students from the University of California, Los Angeles (UCLA) with normal or corrected-to-normal vision were enrolled in the study. Exclusion criteria included seven students who participated in the first session of the study, but either did not show up to the latter session or were unable to be contacted after ~2 months for the second session. In addition, two participants were not included in the analysis due to technical difficulties (that these two participants were not tested the correct action sets for their own-body movements). Hence, a total of 34 participants (21 female, and 13 male) were included in the analyses reported in the present paper.

Participants gave informed consent as approved by the UCLA Institutional Review Board and were provided with $10 cash per session (hence $20 for completing two sessions of the study), except for two participants who requested course credit instead. All participants were naïve to the hypothesis under investigation. Participants were not informed at any point about testing for self-recognition until the final task (i.e., second session) of the study.

Participants completed the 50-point Autism-spectrum Questionnaire (AQ) to generate AQ scores measuring the degree to which individuals with normal intelligence and development show traits associated with the autistic spectrum (Baron-Cohen et al., 2001). Group assignment was determined by degree of autistic traits based on AQ scores, with a cutoff of <15 for the low-AQ group and >24 for the high-AQ group. These cutoff scores were based on previous work in our lab (van Boxtel and Lu, 2013; Thurman et al., 2016), and from previous measurements of these traits in the general population (Ruzich et al., 2015). These cutoffs correspond to the bottom 15th percentile and top 25th percentile of measured AQ histograms from a large sample of 496 UCLA undergraduate students who previously completed the questionnaire in unrelated studies. From this pool of participants with recorded AQ scores, we contacted individuals who were within the cutoff ranges and recruited participants for this study. The high-AQ group consisted of 15 participants (*M*_age_ = 21.3, *SD*_age_ = 1.8, 11 females) with an average AQ score of 26.4 at the 91th percentile (*SD* = 1.5, range = 25–30). The low-AQ group consisted of 19 participants (*M*_age_ = 21.9, *SD*_age_ = 3.2, 10 females) with an average AQ score of 10.1 at the 5th percentile (*SD* = 2.6, range = 4–14).

The sample size of the present study exceeded typical samples (*N* = 6~12) used in previous research studying self-recognition of body movements (Loula et al., 2005; Jokisch et al., 2006). In comparison with sample sizes used in previous studies examining the impact of autism disorder on biological motion perception, the number of high-AQ and low-AQ participants in the present study is within the typical range (*N* = 12 ASD participants in Blake et al., 2003, *N* = 16 in Murphy et al., 2009, *N* = 30 in Koldewyn et al., 2009, and *N* = 16 in van Boxtel et al., 2016). Additionally, in our previous studies examining the relation between autistic traits and adaptation in biological motion, a similar number of students (*N* = 30) participated in a behavioral study (van Boxtel and Lu, 2013), and 12 students participated in an fMRI study (Thurman et al., 2016). Hence, the sample size in the present study is consistent with the participant numbers used in previous studies in the relevant literature.

### Stimuli and Apparatus

**Figure 1:**
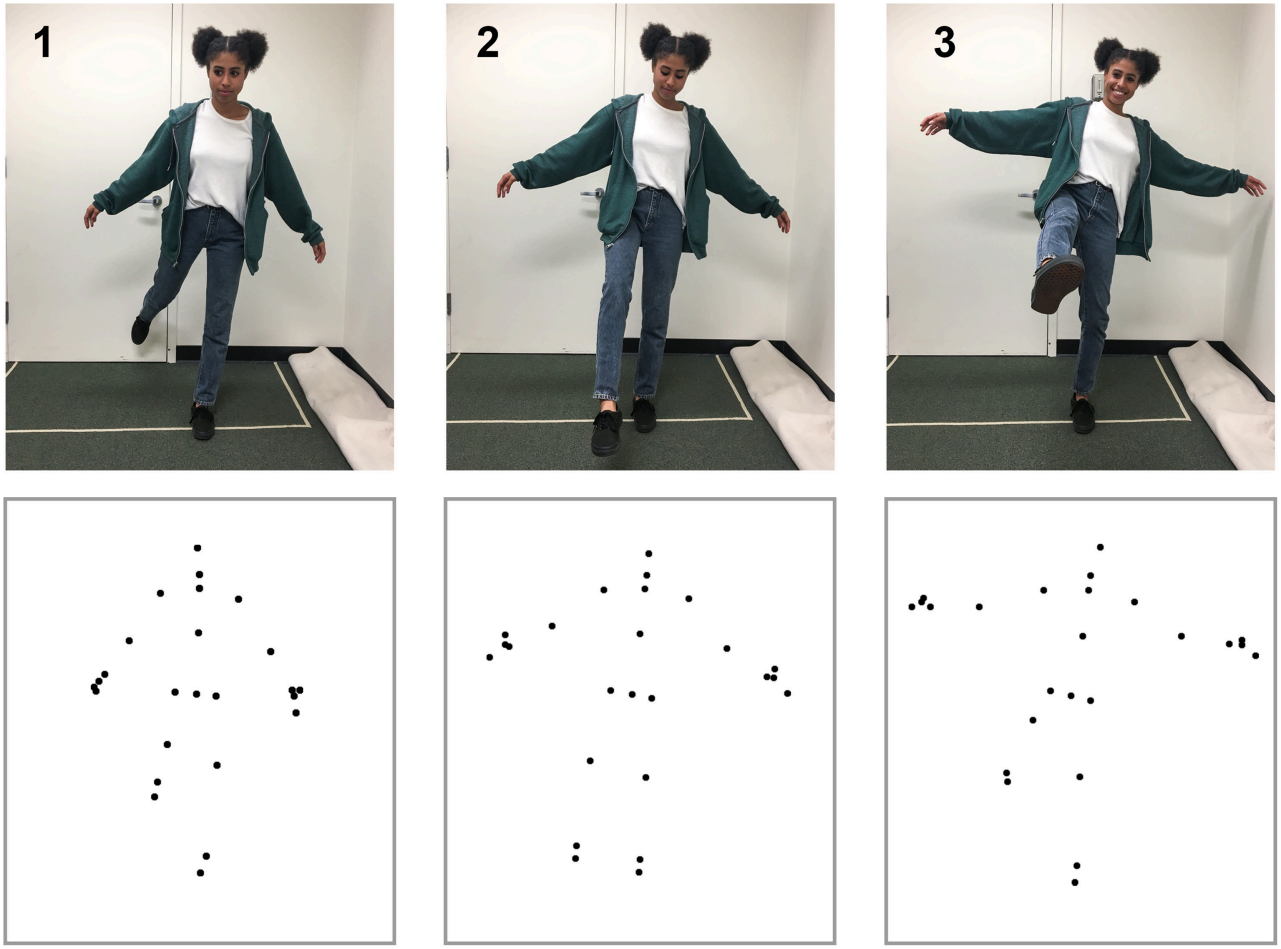
Motion capture data for the simple action kicking. The **top** row shows three image frames from the action sequence, and the **bottom** row shows the corresponding point-light display that was captured from the Kinect device. Not all of the points shown were used for display during the self-recognition task. The depicted individual has given written, informed consent to use her image in the publication.

To create the action stimuli, we used the Microsoft Kinect V2.0 and Kinect SDK (Shotton et al., 2011; Zhang, 2012) to capture three-dimensional coordinates for 18 points (3 points for each limb, 3 for torso, 2 for hands, and 1 for the head) at a rate of 30 Hz. We reduced the noise of movements by applying a double exponential adaptive smoothing filter (LaViola, 2003), which mainly serves to remove recording errors from the Kinect system (e.g., missing a joint due to occlusion or small jitter for some points). We trimmed the videos to present each action segment in point-light display format (van Boxtel and Lu, 2015) for the self-recognition task. Motion capture took place in a 3 by 4 m space to allow for a range of movement. The Kinect sensor was placed 1.2 m above the floor and 2.5 m from the participant. Figure 1 provides an illustration of the action recording process. Testing took place in a dark, quiet room to minimize distractions. Using a chin rest, participants viewed biological motion displays of actions from a fixed distance of 34.5 cm. Each of the 18 dots used for point-light displays subtended a visual angle of 0.15°. Monitor width and height was 53.1° × 40.7°. All videos of the point-light displays can be found at the website “http://cvl.psych.ucla.edu/self_recognition_videos.html.”

### Procedure and Design

Each participant completed two sessions. Motion capture took place in the first session and the self-recognition test in the second session, with a time gap on the average of 2.76 months between the two sessions (in days, *M* = 84.1, *SD* = 31.8, range = 59–154). A long delay between the motion recording session and recognition session has previously been used in a study of self-recognition of body movements (Loula et al., 2005), serving to minimize the likelihood that participants would remember the specific movements that they had performed during the recording session.

We recorded whole-body movements from each participant while they performed a total of 18 different actions. Participants were given verbal instructions, e.g., “please perform the action of grabbing.” This wording was general and did not provide specifics on how to perform the action. Based on previous findings that humans are adept at recognizing various action categories from whole-body movements (Dittrich, 1993; van Boxtel and Lu, 2011), we asked people to perform a variety of actions and later tested self-recognition from a range of actions. We categorized nine stimuli as *simple* actions, and the other nine stimuli as *complex* actions. We categorized actions with simple goals (such as grabbing) or locomotive actions (such as jumping) as *simple* actions, and categorized goal-directed actions (such as arguing) as *complex* actions. In the present study, simple actions consisted of *grabbing, hammering, jumping, kicking, lifting, pointing, punching, pushing*, and *waving*. Complex actions consisted of *arguing, cleaning, dancing, playing a sport, fighting, getting someone's attention, hurrying, playing a musical instrument*, and *stretching*. We imposed a maximum time limit of 5 s for performing each action. Participants were given verbal instructions about performing the actions and asked to think about each action before performing it, in order to avoid spontaneous and unrelated movements. None of the participants showed any problem in understanding the verbal instructions.

The first session of action recording took approximately 45 min. An additional, separate group of six students (three female and three male) were recorded performing the same sets of actions. The recorded actions from these six actors were used solely as distractors during testing in the second session. These six students did not participant in the second session of the study.

**Figure 2:**
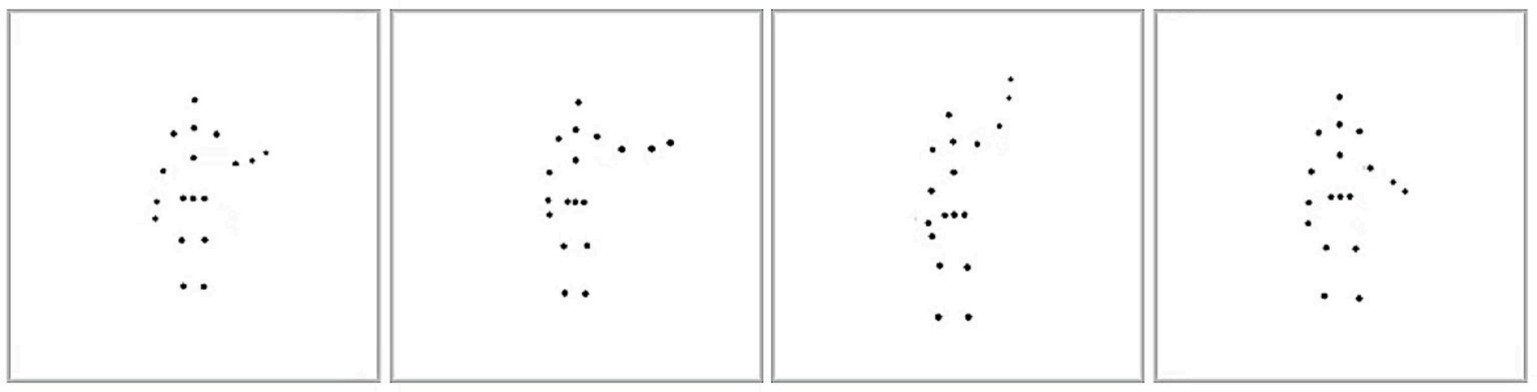
Point-light displays of the self-recognition task. The image shows the subject and three distractor actors performing the waving action.

After a considerable delay (~2.75 months), we measured participants' self-recognition ability during the second session by presenting them with four different point-light stimuli showing the same action performed by four individuals. One point-light stimulus consisted of a participant's own-body movements from previous recordings, and the three others were movements generated by the distractor actors. The distractors matched the participant's gender to avoid gender-specific biases in recognizing body movements. All the displayed action stimuli were normalized according to the participant's body height and width to avoid recognition based on body form. The actions were spread out horizontally along the center of the screen, as shown in Figure 2. Participants were instructed to identify their own action. Each action was looped until the participant made a response by clicking one of the four boxes with the mouse, or until a 40 s timeout. Feedback was not provided. Within each trial, all four point-light stimuli were displayed from the same viewing angle. In the test session, four viewpoints were used to show each action. Specifically, the actors were rotated around the vertical axis to show the action at 0° (facing back), 135° (facing front right), 180° (facing front), and 247° (facing back left). We did not use profile views (directly facing left or right), since some actions (e.g., jumping) induced severe self-occlusion of moving point-lights at these views. There were 72 test trials in total in the self-recognition session (four viewpoints for each of the 18 actions).

In addition to measuring self-recognition accuracy, we recorded participants' response times and confidence in their self-recognition judgments. For confidence assessment, we adopted the design of comparative confidence judgments (De Gardelle and Mamassian, 2014). Trial order was constrained so that every two trials participants viewed one simple action and one complex action. The order and selection of actions was randomized. After viewing two self-recognition trials, participants were asked to indicate in which trial they were more confident in their decision of identifying own-body movements. Specifically, after two trials of self-identification, participants were shown two side-by-side boxes, with box “1” indicating they were more confident in their judgment for the first of the previous two trials, and “2” for more confidence in their judgment for the second of the previous two trials. There were 36 confidence measurements in total.

## Results

Overall self-recognition accuracy (proportion correct) for each of the 18 actions (including all participants) was in the range of [0.43, 0.86], significantly above the chance level of 0.25. This result replicated findings from prior research showing that humans are able to identify themselves from walking actions (Cutting and Kozlowski, 1977; Loula et al., 2005; Jokisch et al., 2006). Hence, the present findings provide converging evidence that participants are able to recognize themselves solely from kinematic information in body movements, even for actions less commonly encountered than walking.

**Figure 3:**
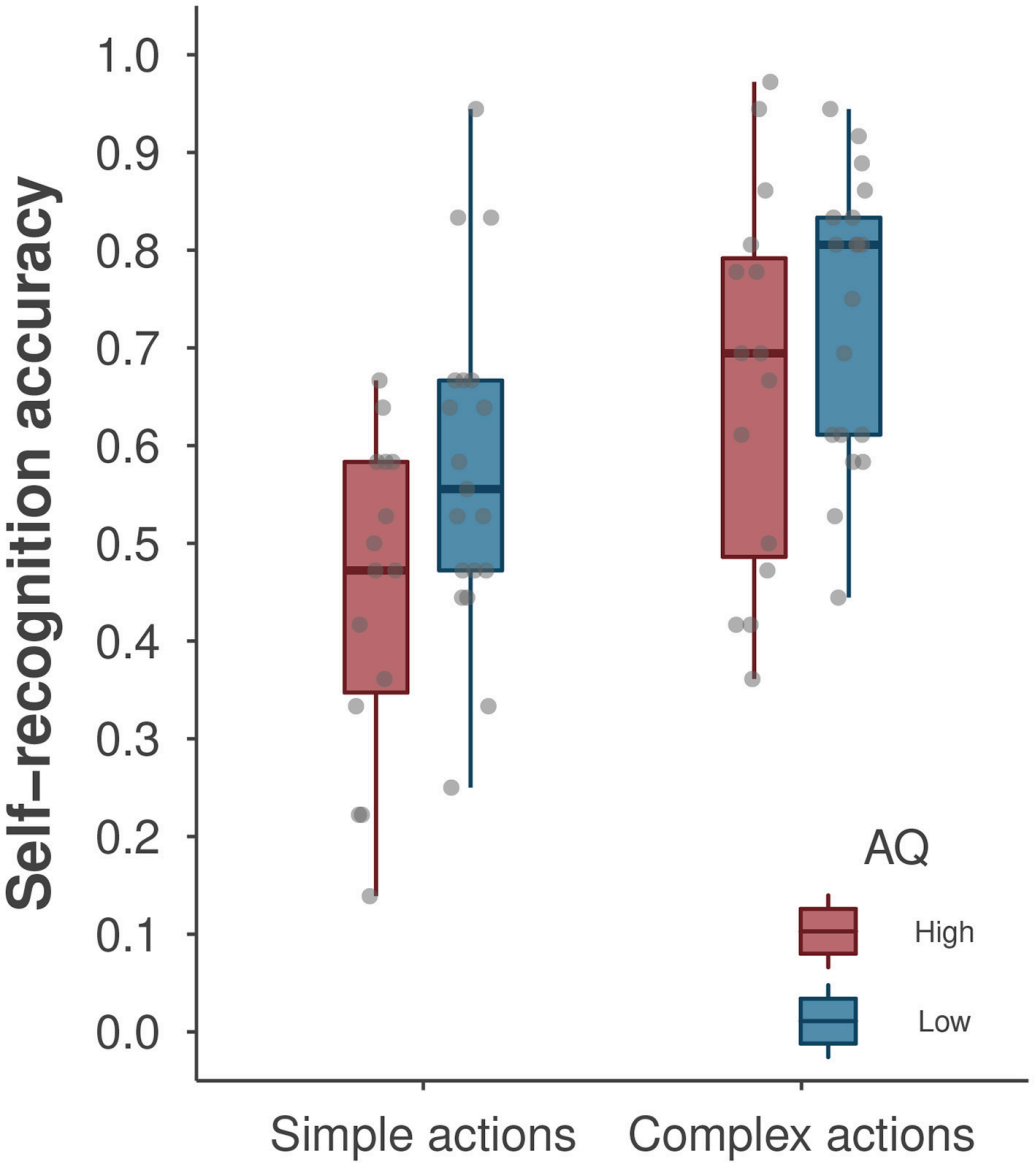
A boxplot of self-recognition accuracy by AQ group and action type. The boxplot shows the median accuracy and the first/third quartiles. The points denote the average accuracy of individual participants. Note that data points for participants yielding the same accuracy level may overlap in the plot.

We first conducted a repeated-measures ANOVA to examine the effects of AQ group (low vs. high, a between-subjects factor) and action type (simple vs. complex, a within-subjects factor) on self-recognition performance. Figure 3 depicts the average self-recognition accuracy as a function of action complexity and AQ groups. We found a significant main effect of action type [*F*_(1, 32)_ = 40.59, *p* < 0.001, η_*p*_^2^ = 0.56], with better self-recognition performance for complex than for simple actions. This analysis revealed a marginal main effect of AQ group [*F*_(1, 32)_ = 3.80, *p* = 0.060, η_*p*_^2^ = 0.11], suggesting that the degree of autistic traits may impact self-recognition performance. The low-AQ group performed significantly better at self-recognition of simple actions than did the high-AQ group [*t*_(32)_ = 2.23, *p* = 0.033, η_*p*_^2^ = 0.13], but these group differences were not observed for complex actions [*t*_(32)_ = 1.34, *p* = 0.256, η_*p*_^2^ = 0.04]. The classic repeated-measures ANOVA did not reveal a significant interaction effect between AQ group and action type [*F*_(1, 32)_ = 1.12, *p* = 0.298].

**Figure 4:**
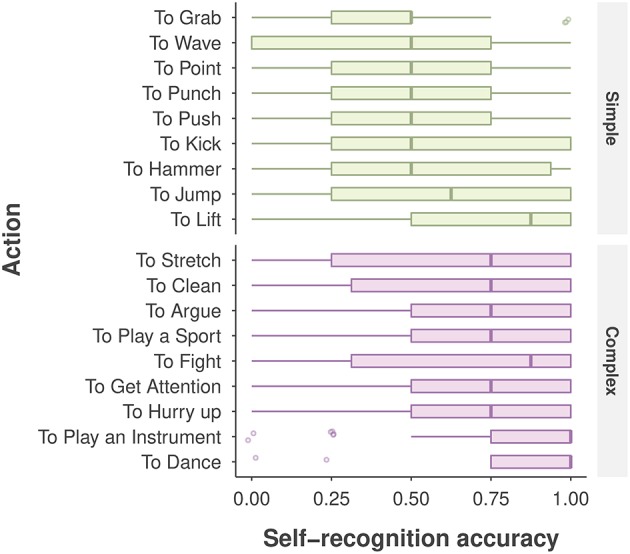
Self-recognition accuracy for each action, categorized by action type. The boxplot displays the median (the central vertical line), the lower 25th and upper 75th percentiles (the left and right edges of the box), and whiskers (box edge ±1.5 × box width). The points denote the outliers (any value beyond the whiskers).

As depicted in Figure 4, participants showed large variability in self-recognition accuracy for individual action stimuli. For example, accuracy was 0.86 for the *dancing* action, but was 0.65 for the *stretching* action, despite the fact that both actions were categorized as complex; and for the two simple actions *lift* and *grab*, performance was 0.72 and 0.43, respectively. Such large variability involving individual actions, viewpoints, and other extraneous variables such as gender, age, and AQ score were not captured in the aforementioned ANOVA analysis.

To take into consideration these potential sources of variability involving individual actions and participants, we conducted a Bayesian analysis of a hierarchical logistic regression model with a binary response (correct/incorrect) for each trial as the dependent measure (Carpenter et al., 2017). The hierarchical model included predictor variables of fixed effects, varying predictors at the participant level, and varying predictors at the action level. We compared three different models to first identify the important factors for determining self-recognition accuracy. The full model, Model 1, included fixed effects action type (simple vs. complex), AQ group (low vs. high), and the interaction between action type and AQ group. Predictors at the participant level included random intercepts and action type factors. Predictors at the action level included random intercepts and viewpoints. We also included participant age, gender, and AQ score as group-level predictors.

We compared Model 1 with two alternative models. Model 2 excluded the interaction effect of action type by AQ group but kept everything else the same, and Model 3 excluded action type and AQ factors. The three models were compared using approximate leave-one-out cross validation (LOO) with the Pareto-smoothed importance sampling (PSIS) method (Vehtari et al., 2017). Specifically, PSIS-LOO is based on the expected log predictive density while also penalizing the number of estimated parameters. This method is considered an improvement over the Deviance Information Criterion (DIC) for Bayesian models. The Bayesian model comparison revealed that Model 1 provided a better fit to human performance than did the two alternative models, according to the obtained LOO values: Model 1 vs. Model 2 resulted in ΔLOO = 15.9, SE = 5.6, CI = [4.9, 27.0]; Model 1 vs. Model 3 resulted in ΔLOO = 17.6, SE = 6.0, CI = [5.9, 29.3]. The results from the Bayesian analysis indicated that after considering variability from extraneous variables (e.g., individual actions, viewpoints), AQ group, action complexity, and the interaction between these two factors all play important roles in accounting for human performance for self-recognition of body movements. Details of the Bayesian analysis are included in the Supplementary Materials.

**Figure 5:**
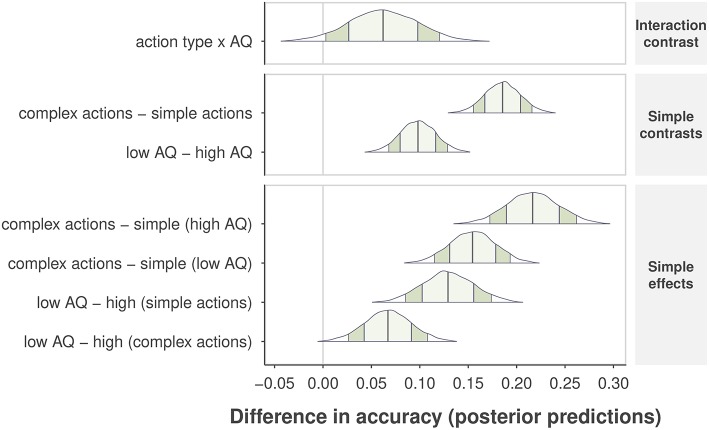
Differences in posterior predictive accuracy are shown for specific sets of contrasts and effects along the vertical axis. Posterior predictions are determined from the observed data and by comparing the mean predicted value at specific levels of one or more factors. The “no difference” marker is indicated by the vertical line at value zero. The shape of the posterior distribution is shown for each contrast, with 90% intervals indicated by the darker shaded region inside the distribution, and 1 SD of the posterior by the lighter, center shaded region. Posterior means are indicated by the center vertical lines inside each distribution.

Next, we selected Model 1 to obtain the posterior *distributions* of predicted accuracy for the effects of AQ group and action complexity. We report 90% Bayesian posterior confidence intervals for each effect (Morey et al., 2016), and *p*-values indicating how much of the posterior distribution is above the null threshold (i.e., lack of a difference within a single contrast comparison). The Bayesian analysis confirmed that complex actions were easier to identify than simple actions, resulting in an increase in accuracy of .19, CI = [0.15, .22], *p* < 0.001, as shown in the second row plot in Figure 5. Due to the consideration of variability at the levels of subjects and individual actions, the Bayesian analysis gained more power to reveal a main effect of self-recognition accuracy between AQ groups, indicating that the high-AQ group was less accurate than the low-AQ group by 0.10, CI = [0.07, 0.13], *p* < 0.001, as shown in the third row plot in Figure 5. We also found an interaction effect, such that the impact of AQ group on performance decreased from complex to simple actions. High-AQ participants showed a further reduction in accuracy of 0.06, CI = [0.003, 0.12], *p* = 0.044 compared to low-AQ participants. These results indicate that individuals with higher levels of autistic traits show worse performance when identifying themselves from *simple* actions in comparison with low-AQ individuals. We observed no difference in accuracy between males and females in the high-AQ group, but found that males were 0.07, CI = [0.04, 0.12] more accurate than females in the low-AQ group.

We also examined the results from the other two dependent measures, response time (RT) and confidence. For the RT analysis, we removed 10 trials from the total of 2,448 trials (34 participants × 72 trials) in which eight of the participants did not respond within 40 s (these trials were counted as inaccurate for the analysis based on accuracy data). We fit the same effects as in Model 1, but with the normalized log response time as the outcome and a Gaussian distribution as the likelihood function. Response time patterns showed similar significant main effects to those observed for accuracy data. The high-AQ group responded 5.5 (*p* < 0.001) s faster than did the low-AQ group, and simple actions yielded a median RT 6.1 s shorter than that for complex actions (*p* < 0.001). However, the RT data did not show the interaction effect between AQ group and action type (see the Supplementary Materials for posterior medians of each contrast effect for the RT analysis). However, we emphasize that accuracy was the primary measure in this study, and the relatively low overall accuracy levels make it more difficult to interpret the RT data.

Finally, for the confidence measure, we computed the proportion of complex action selections over simple action selections and analyzed these proportions by AQ group. The mean proportion for complex action confidence was 0.612 (*SE* = 0.04) for the low AQ group, and 0.609 (*SE* = 0.03) for the high AQ group. There was no significant group difference between the two AQ groups. This finding suggests that participants were more confident in their choice of own-body movements for complex actions than for simple ones, regardless of the degree of autistic traits.

## Discussion

Although humans rarely witness own-body movements, people can still identify themselves solely based on the kinematics of these movements, even when reduced to sparse visual information and scant self-identifying cues provided by point-light stimuli. We replicated findings from prior research showing that humans are able to identify themselves from walking actions (Cutting and Kozlowski, 1977; Loula et al., 2005; Jokisch et al., 2006). Since the present study used a variety of actions, our results generalize previous findings by showing that participants can recognize themselves solely from body movements even for actions less familiar than walking.

The present study is the first study to report individual differences in the ability of self-recognition with body movements. We found clear evidence that human performance of self-recognition is influenced both by characteristics of extrinsic stimuli such as complexity of performed actions, and by intrinsic traits of observers such as autistic traits. Our results revealed significantly better self-recognition performance for complex actions than for simple actions. This impact of action complexity may result from the increased motor planning and the greater individualization of movements involved in performing complex actions. For example, different people may generate different movements for a complex action such as *getting someone's attention*. Although simple actions (e.g., walking, jumping) are commonly observed and performed in daily life, biometric identity cues may not be readily apparent to the human visual system, resulting in a difficulty to differentiate these actions of individuals and in self-identification.

Intrinsic traits of observers can also affect the ability of self-recognition of body movements. A key finding in the present study is that participants with a high degree of autistic traits showed poorer performance for self-recognition of body movements than did people with fewer autistic traits. This finding is consistent with previous findings that individuals with autism show impairments on various tasks involving motor skills and social perception (e.g., Bailey et al., 1995; Rinehart et al., 2006; Kaiser et al., 2010; Ahmed and Vander Wyk, 2013; van Boxtel et al., 2016). What underlying mechanisms may contribute to the impact of autistic traits on self-recognition of body movements? We discuss a few possibilities below.

It is well documented that autism is associated with motor skill impairments (e.g., Rinehart et al., 2006; Ming et al., 2007). The impact of autistic traits on self-recognition performance in our study may result from some differences in motor movements between the high-AQ and the low-AQ groups. To examine this possibility, we compared a general characteristic of motor movements to assess whether people with high AQ scores moved faster or slower than people with low AQ scores. We compared the average speed of movements between the two groups. Using the recorded 3D coordinates of motion capture data from participants in the study, we calculated the inter-frame displacements for each point. We then computed the average speed of movements across the frames and points for each action, and compared the movement speeds between the high-AQ and the low-AQ groups. Only for the *jumping* action, people in the high-AQ group moved significantly slower than people in the low-AQ group (mean movement speed: high-AQ group 0.7 inch per frame; low-AQ group 1.1 inch per frame; *p* = 0.01). The other 17 of the 18 actions did not reveal any speed difference between the two groups. Hence, we found no clear evidence that the two AQ groups performed actions differently in a systematic way, which weakens the possibility that motor skill differences between two AQ groups can account for the present findings.

Since we rarely see our own body movements, self-recognition of actions need not be solely vision-based, more likely relying on the linkage between action perception and the motor processing system. Motor representations of own-body movements must be converted to visual representations that could be used in the self-recognition task. It is possible that people with a high degree of autistic traits may have a weakened connection between visual and motor representations of body movements, resulting in decreased self-recognition performance for the high-AQ group. One possible explanatory mechanism is related to the mirror neuron network, which includes unique neurons that discharge both when an action is perceived and when it is executed (Gallese et al., 1996; Rizzolatti et al., 1996; Rizzolatti and Fabbri-Destro, 2010). Mirror neurons may play a critical role in a coding scheme for the “self” and “other,” serving to transform a motor representation of own-body movements into a visual representation. Accordingly, a weakened mirror neuron network in the high-AQ group may have led to poorer performance in self-recognition of body movements.

This conjecture is consistent with the proposal by Williams (2008), specifically that a deficient mirror neuron system may give rise to impairments in self-other matching ability for individuals with ASD. However, the possibility of mirror- neuron abnormalities in autism remains controversial and is an active research area. Some research suggests that deficits in the development of mirror neuron networks in children with ASD may lie at the root of perceptual and social behavior impairments (Pineda, 2005; Dapretto et al., 2006; Le Bel et al., 2009). However, others have suggested that impairments in other brain systems beyond the mirror neuron system are the causes of poor social ability in autism (see a review by Southgate and Hamilton, 2008). Future work is clearly needed to pin down the relation between the mirror system and self-recognition ability. Currently, the experimental paradigm developed in the present paper provides a new stimulus set that could be utilized to investigate the mirror system in autism. In addition, the Kinect motion capture system makes it much more feasible to film complex actions performed by children with autism. Although the Kinect system does not have as high precision as other motion capture systems, the present study showed that the system has adequate recording quality to support the self-recognition task.

Finally, it is also possible that the poorer self-recognition by high-AQ individuals in our study may result from less attention to social stimuli. According to the social motivation theory of autism proposed by Chevallier et al. (2012), a dysfunction of social motivational mechanisms may constitute a primary deficit in autism and may lead to an unbalanced tradeoff between attention to external cues and attention to internal cues. For example, individuals with ASD are less susceptible to the rubber-hand illusion and show disproportionate attention to internal body cues (Schauder et al., 2015). This reduced attention and social motivation may be linked to impairments in the early stages of action encoding, which can subsequently affect kinematic memory (Zalla et al., 2010) and weaken top-down mechanisms involved in biological motion perception (Lu et al., 2006).

The construct of a “self” is highly complex, involving several subcomponents such as awareness of oneself (and consequently one's own actions), a sense of agency, and body ownership (Van Den Bos and Jeannerod, 2002). The ability to self-recognize is a necessary subcomponent of human social interaction and communication, helping to establish oneself as an independent agent (Jeannerod, 2003). The apparent ease and automaticity of self-recognition belies a highly complex integrative process, in which a conscious self-representation stems from the interplay between perceptual and motor systems. Extending self-recognition to own-body movements highlights the important contribution of motor experience to self-recognition, providing a new experimental paradigm to examine the core construct of the “self.”

## Author Contributions

Experimental design contributed by HL and JB. Data collection and data processing contributed by TS and JB. Data analysis and manuscript preparation contributed by JB, HL, and AK.

### Conflict of Interest Statement

The authors declare that the research was conducted in the absence of any commercial or financial relationships that could be construed as a potential conflict of interest.
