# Supplementary material for: The Impact of Autistic Traits on Self-Recognition of Body Movements
Source: Front Psychol. 2019 Jan 10;9:2687. doi: 10.3389/fpsyg.2018.02687 (PMC6338035; doi:10.3389/fpsyg.2018.02687)
Supplement: Supplementary file 1 [file Data_Sheet_1.PDF]

Supplementary Material: The Impact of Autistic Traits on Self-Recognition of Body Movements

1 INTRODUCTION

An up-to-date version of this document and all supplementary materials can be found at the following repository.

https://github.com/iamamutt/SRE

The data can be obtained without installing the package. See `data/SRE.rda` or `inst/extdata/sre.csv` for a text version. Please review the `README.md` file for package installation instructions and how to use the exported R package functions.

1.1 Dataset

The dataset has 2448 rows and contains the following variables. Displayed are the column names, variable types, and a sample of their values.

- `id` (*integer*): [5, 24, 21, ...]
- `exp_date` (*character*): [03-Aug-2016, 04-Apr-2016, 04-Feb-2016, ...]
- `sub_age` (*numeric*): [21, 21, 35, ...]
- `sub_sex` (*factor*): [f, f, m, ...]
- `aq_score` (*integer*): [25, 11, 10, ...]
- `aq_category` (*factor*): [high symptoms, low symptoms, low symptoms, ...]
- `block` (*integer*): [1, 1, 2, ...]
- `trial` (*integer*): [1, 2, 3, ...]
- `action_category` (*factor*): [simple actions, complex actions, complex actions, ...]
- `action` (*factor*): [To Jump, To Stretch, To Fight, ...]
- `angle` (*factor*): [247, 180, 0, ...]
- `recog_acc` (*integer*): [1, 0, 1, ...]
- `recog_rt` (*numeric*): [11, 19, 21, ...]
- `conf_choice` (*integer*): [1, 1, 1, ...]
- `conf_rt` (*numeric*): [3.3, 2.2, 8.7, ...]
- `recog_corr_box` (*factor*): [4, 3, 4, ...]
- `recog_resp_box` (*factor*): [4, 2, 4, ...]
- `days_elapsed` (*integer*): [148, 75, 79, ...]
- `sub_age_scaled` (*numeric*): [0.41, 0.38, 3, ...]
- `aq_score_scaled` (*numeric*): [0.96, 0.13, 0.067, ...]
- `recog_rt_scaled` (*numeric*): [0.58, 1.2, 1.4, ...]
- `trial_scaled` (*numeric*): [-0.35, -0.33, -0.31, ...]

- `aq_score_standard` (*numeric*): [`1.2`, `-0.031`, `-0.12`, ...]

2 SUMMARY STATISTICS

The current section contains tables of summary statistics computed from the package dataset `SRE`.

2.1 Subject pool statistics

Information regarding the participant sample, such as their AQ scores, age, sex, and testing delay time is displayed below.

2.1.1 Participant age

Table S1: Participant age by AQ groups.

AQ	N	mean	sd	min	max
High	15	21.3	1.8	18.7	26.7
Low	19	21.9	3.23	19.6	34.7
.	34	21.6	2.68	18.7	34.7

2.1.2 Test delay

Table S2: Delay between recording and test (days).

AQ	N	mean	sd	min	max
High	15	74.267	29.361	59	148
Low	19	91.842	32.222	61	154
.	34	84.088	31.788	59	154

2.1.3 AQ scores

Table S3: AQ score by AQ groups.

AQ	N	mean	sd	min	max
High	15	26.4	1.5	25	30
Low	19	10.1	2.63	4	14
.	34	17.3	8.52	4	30

Table S4: AQ score by sex.

Sex	N	mean	sd	min	max
F	21	18.8	8.29	8	30
M	13	14.8	8.61	4	29
.	34	17.3	8.52	4	30

Table S5: AQ score by AQ groups and sex.

Sex	AQ	N	mean	sd	min	max
F	High	11	26.4	1.5	25	30
F	Low	10	10.5	1.96	8	13
M	High	4	26.5	1.73	25	29
M	Low	9	9.56	3.28	4	14
.	.	34	17.3	8.52	4	30

2.2 Accuracy summary statistics

Summary statistics from the self-recognition accuracy column (`recog_acc` from the `SRE` dataset) are displayed below.

2.2.1 Accuracy for each subject, split by AQ group

Table S6: Accuracy by subject.

AQ	id	N	mean	sd	min	max
High	8	72	0.81	0.4	0.29	0.81
High	14	72	0.72	0.45	0.29	0.81
High	29	72	0.72	0.45	0.29	0.81
High	19	72	0.71	0.46	0.29	0.81
High	9	72	0.69	0.46	0.29	0.81
High	26	72	0.65	0.48	0.29	0.81
High	32	72	0.64	0.48	0.29	0.81
High	2	72	0.57	0.5	0.29	0.81
High	5	72	0.53	0.5	0.29	0.81
High	13	72	0.5	0.5	0.29	0.81
High	12	72	0.42	0.5	0.29	0.81
High	22	72	0.38	0.49	0.29	0.81
High	31	72	0.38	0.49	0.29	0.81
High	10	72	0.35	0.48	0.29	0.81
High	1	72	0.29	0.46	0.29	0.81

Table S7: Accuracy by subject.

AQ	id	N	mean	sd	min	max
Low	18	72	0.88	0.33	0.39	0.88
Low	7	72	0.88	0.33	0.39	0.88
Low	21	72	0.83	0.38	0.39	0.88
Low	33	72	0.76	0.43	0.39	0.88
Low	23	72	0.72	0.45	0.39	0.88
Low	27	72	0.72	0.45	0.39	0.88
Low	11	72	0.67	0.47	0.39	0.88
Low	17	72	0.65	0.48	0.39	0.88
Low	24	72	0.64	0.48	0.39	0.88
Low	3	72	0.64	0.48	0.39	0.88
Low	28	72	0.64	0.48	0.39	0.88
Low	6	72	0.64	0.48	0.39	0.88
Low	15	72	0.62	0.49	0.39	0.88
Low	16	72	0.62	0.49	0.39	0.88
Low	20	72	0.61	0.49	0.39	0.88
Low	34	72	0.6	0.49	0.39	0.88
Low	4	72	0.51	0.5	0.39	0.88
Low	30	72	0.42	0.5	0.39	0.88
Low	25	72	0.39	0.49	0.39	0.88

2.2.2 Accuracy for each action

Table S8: Accuracy by action.

	Actions	N	mean	sd
Complex	To Dance	136	0.86	0.35
Complex	To Play an Instrument	136	0.79	0.41
Complex	To Hurry up	136	0.69	0.46
Complex	To Get Attention	136	0.68	0.47
Complex	To Fight	136	0.68	0.47
Complex	To Play a Sport	136	0.66	0.47
Complex	To Argue	136	0.66	0.47
Complex	To Clean	136	0.65	0.48
Complex	To Stretch	136	0.65	0.48
Simple	To Lift	136	0.72	0.45
Simple	To Jump	136	0.57	0.5
Simple	To Hammer	136	0.54	0.5
Simple	To Kick	136	0.54	0.5
Simple	To Push	136	0.53	0.5

	Actions	N	mean	sd
Simple	To Punch	136	0.46	0.5
Simple	To Point	136	0.44	0.5
Simple	To Wave	136	0.44	0.5
Simple	To Grab	136	0.43	0.5

2.2.3 Accuracy by participant sex

Table S9: Accuracy by sex.

Sex	AQ	N	mean	sd	min	max
F	Low	720	0.62	0.49	0.55	0.62
F	High	792	0.55	0.5	0.55	0.62
M	Low	648	0.7	0.46	0.58	0.7
M	High	288	0.58	0.49	0.58	0.7
F	.	1512	0.58	0.49		
M	.	936	0.66	0.47		

3 BAYESIAN HIERARCHICAL LOGISTIC REGRESSION MODELS

3.1 Model Specification

3.1.1 Binomial Likelihood

We model participants' responses as a binary random variable, each participant providing a vector of values $\mathbf{y}$ with either 1 for correct or 0 for incorrect. The model-predicted binary values $\hat{\mathbf{y}}$ of the observed responses $\mathbf{y}$ are the result of (1) applying an inverse link function g^{-1} to the linear predictor $\boldsymbol{\eta}$, providing a probability vector $\tilde{y}$ of real values constrained between 0 and 1, and (2) using a binary step activation function f to map the probability vector back to a binary response vector.

$$\begin{aligned}
\mathbf{y} &= \hat{\mathbf{y}} + \boldsymbol{\epsilon} \\
\hat{\mathbf{y}} &= f(\tilde{\mathbf{y}}) \\
\tilde{\mathbf{y}} &= g^{-1}(\boldsymbol{\eta}) \\
\boldsymbol{\eta} &= \mathbf{X}\boldsymbol{\alpha} + \mathbf{Z}\boldsymbol{\beta}
\end{aligned}$$

The linear predictor $\boldsymbol{\eta}$ is on the logit scale and takes the form of a regression equation with its input as variables of interest and their estimated coefficients. Generalized linear models use a link function g to map the linear predictor to the dependent variable. If the response variable is Gaussian distributed, then the link and activation functions are simply the identity function.

$$\text{Identity} : g(x) = f(x) = x$$

To map logit scaled values to a probability vector constrained between 0 and 1 we use the inverse logit link function (sigmoid).

$$g^{-1}(\eta) = \frac{1}{1 + e^{-\eta}}$$

The binary step activation function creates the binary response vector $\hat{\mathbf{y}}$ and is a simple thresholding operation at equal probability.

$$f(\tilde{y}) = \begin{cases} 0 & \text{for } \tilde{y} < 0.5 \\ 1 & \text{for } \tilde{y} \geq 0.5 \end{cases}$$

Thus, the combined steps from linear predictor value η_i to predicted response $\hat{y}_i$ are

$$\hat{y}_i = f(g^{-1}(\eta_i))$$

The general form of a Bernoulli trial with n number of trials is a Binomial distribution with the probability mass function,

$$\binom{n}{y} \tilde{y}^y (1 - \tilde{y})^{n-y}$$
$$\tilde{y} = g^{-1}(\eta)$$

Table S10: Log-likelihoods of getting none to all four responses correct for a specific action, given that there are four options to choose for each trial.

0	1	2	3	4
-1.1507	-0.86305	-1.5562	-3.0603	-5.5452

Predictions on the logit scale are estimated from the data matrices $\mathbf{X}$ and $\mathbf{Z}$, and their corresponding coefficients $\boldsymbol{\alpha}$ and $\boldsymbol{\beta}$.

$$\hat{y}_i = X_i\boldsymbol{\alpha} + Z_i\boldsymbol{\beta}$$

Individual deviations from the mean predicted values (vector ϵ) are referred to here as *error*, and together make up the observed values of y.

$$\mathbf{y} = \hat{\mathbf{y}} + \boldsymbol{\epsilon}$$

No-U-Turn, a variant of the Hamiltonian Monte Carlo algorithm that automates the algorithms parameters, converges to the target probability space for the estimated parameters by avoiding random walks associated with Metropolis-Hastings or Gibbs sampling (Carpenter et al., 2017; Homan & Gelman, 2014). MCMC algorithms used to estimate model parameters condition on the data using Bayes rule. Denoting $\boldsymbol{\Theta}$ to refer to the collection of parameters being estimated, then

$$p(\boldsymbol{\Theta}|\mathbf{y}) = \frac{p(\mathbf{y}|\boldsymbol{\Theta})p(\boldsymbol{\Theta})}{p(\mathbf{y})}$$

The normalizing constant in the denominator of the right-hand side of the equation is ignored given that the numerator is proportional to the left-hand side of the equation. The $p(y|\theta)$ portion from the equation above is the likelihood component, and the prior component, assumptions about how the parameters are distributed, consists of the $p(\theta)$ portion.

3.1.2 Priors

Further breaking down the priors component $p(\theta)$, we specify the distributions of the parameter vectors $\boldsymbol{\alpha}$ and $\boldsymbol{\beta}$. $\boldsymbol{\alpha}$ is a single column-vector of parameters that account for the observation-level fixed effects (unmodeled, responses at the trial level), which do not vary by group. These are mapped to the fixed effects design matrix $\mathbf{X}$. They don't include an intercept term (α_0), since this is estimated separately to avoid unidentifiability issues across multiple levels of a hierarchical model.

$$\boldsymbol{\alpha} = \alpha_1, \ldots, \alpha_p$$

The $\boldsymbol{\alpha}$ coefficients are given a student's t distribution with degrees of freedom parameter ν, mean μ, and standard deviation σ. The t prior has fat tails and is robust to estimating outlier coefficients without over-inflating the standard deviation estimate in order to capture these coefficients (Ghosh, Li, & Mitra, 2015). Assuming that the variables have been rescaled to where X is on the unit scale (most values range between 0 and 1), then the following values for the parameters are used

$$\boldsymbol{\alpha} \sim t(4, 0, 2.5)$$

The average predicted value is estimated by the intercept term and is also given a t prior, but with a standard deviation of 10.

$$\boldsymbol{\alpha_0} \sim t(4, 0, 10)$$

The vector $\boldsymbol{\beta}$ is a set of parameters for every group member (e.g., a random sample of participants or items in an experiment) that maps to a sparse random effects matrix $\mathbf{Z}$. If there are K parameters to be estimated for every member within a group with J members, then there are $J * K$ parameters to be estimated for $\boldsymbol{\beta}$. The parameters $\boldsymbol{\beta}$ are coerced into a vector from an original $J \times K$ matrix $\hat{\boldsymbol{\beta}}$ by transposing, traversing columns, and stacking the values into a single vector.

$$\boldsymbol{\beta} = \text{vectorize}(\hat{\boldsymbol{\beta}}')$$

The $\hat{\boldsymbol{\beta}}$ matrix is sampled from a Multivariate Normal distribution with mean vector $\mathbf{U}_j\boldsymbol{\gamma}$ and covariance matrix $\boldsymbol{\Sigma}$. Each row $\mathbf{U}_j$ for all J make up the matrix $\mathbf{U}$, and is a $J \times L$ design matrix of the group-level fixed effects (similar to X, but predictors specific to groups), with L being the number of group-level predictors (including the intercept term).

$$\hat{\boldsymbol{\beta}}_j \sim \mathcal{N}(\mathbf{U}_j\boldsymbol{\gamma}, \boldsymbol{\Sigma})$$

The matrix $\boldsymbol{\gamma}$ is an $L \times K$ matrix of group level regression coefficients with each row having their own regression equation of group-level fixed effects. These coefficients are also given t priors.

$$\boldsymbol{\gamma} \sim t(4, 0, 2.5)$$

The covariance matrix $\boldsymbol{\Sigma}$ is derived from multiple components, each with their own set of priors. First, we start by estimating the standard deviation vector $\boldsymbol{\sigma}_\beta$. This ultimately specifies the scale of the varying coefficients in $\hat{\boldsymbol{\beta}}$. We also estimate a separate correlation matrix Ω that determines how the $\hat{\boldsymbol{\beta}}$ coefficients vary in relation to each other. With these two components we can compute the covariance matrix associated with $\hat{\boldsymbol{\beta}}$.

$$\boldsymbol{\Sigma} = \text{diag}(\boldsymbol{\sigma}_\beta)\,\boldsymbol{\Omega}\ \text{diag}(\boldsymbol{\sigma}_\beta)$$

The standard deviation vector $\boldsymbol{\sigma}_\beta$ from the covariance matrix $\boldsymbol{\Sigma}$ is composed of the average variance τ^2, and a partitioning vector $\boldsymbol{\pi}$ (unit simplex) that determines the proportion of total variance among the set of K predictors. The sum of the diagonal elements (the trace) of $\boldsymbol{\Sigma}$ is $K\tau^2$.

$$\boldsymbol{\sigma}_\beta = K\tau^2\boldsymbol{\pi}$$

The average standard deviation τ is a scalar value and is given a Gamma distribution with some priors on the shape and scale parameters. These influence the overall scale of the covariance matrix, but are typically used to control the bias of the variances, pushing them closer to or further away from zero.

$$
\begin{aligned}
\tau &\sim \text{Gamma}(\tau_a, \tau_b) \\
\tau_a &= 1 \\
\tau_b &= 1
\end{aligned}
$$

The variance partitioning vector $\boldsymbol{\pi}$ sums to 1, and can be generated from a Dirichlet distribution given a set of concentration parameters $\boldsymbol{\theta}$.

$$\boldsymbol{\pi} \sim \text{Dirichlet}(\boldsymbol{\theta}) = \pi_{1,\ldots,K} \sim \text{Dirichlet}(\theta_{1,\ldots,K})$$

The concentration parameters $\boldsymbol{\theta}$ control the spread of the variances in $\boldsymbol{\sigma}^2_\beta$, and themselves are given a Gamma distribution with some concentration prior ζ for the shape parameter and scale set to 1. If $\zeta < 1$, then variances are largely heterogeneous. When $\zeta = 1$, variance proportions are uniform (no prior information), and when $\zeta > 1$, variances are increasingly homogeneous.

$$
\begin{aligned}
\boldsymbol{\theta} &\sim \text{Gamma}(\zeta, 1) \\
\zeta &= 1
\end{aligned}
$$

If the shape and scale parameters from a Gamma distribution are constants then using $\boldsymbol{\theta}$ for sampling from the Dirichlet distribution can be skipped and instead,

$$\boldsymbol{\pi} = \frac{\boldsymbol{\theta}}{\sum_{k=1}^{K} \theta_k}$$

Lastly, the correlation matrix is given a LKJ prior (estimated from several Beta distributions, see Lewandowski, Kurowicka, & Joe, 2009) which takes a single parameter η (not to be confused with the linear predictor). The parameter η is a regularization term, which controls the strength of correlations among all pairwise predictors. If $\eta > 1$, then stronger correlations are less likely by constraining the diagonal elements to be "peaked", or maintain larger values relative to the off-diagonal elements. If $\eta < 1$, the values on the off-diagonal become larger and stronger correlations are more likely. A value of 1 is Uniformly distributed over all possible correlation matrices, and a value of 2 corresponds to $L2$ regularization.

$$
\begin{aligned}
\boldsymbol{\Omega} &\sim \text{LKJcorr}(\eta) \\
\eta &= 2
\end{aligned}
$$

3.2 MCMC convergence information

Trace plots of each MCMC chain (to ensure samples are not stuck within some region of the target distribution), $\hat{R}$ statistics for gauging chain divergence ($\hat{R} = 1$ is best), and the effective number of samples from the total sample size (a ratio of 1 is best) are shown.

3.2.1 Model 1 convergence diagnostics plots

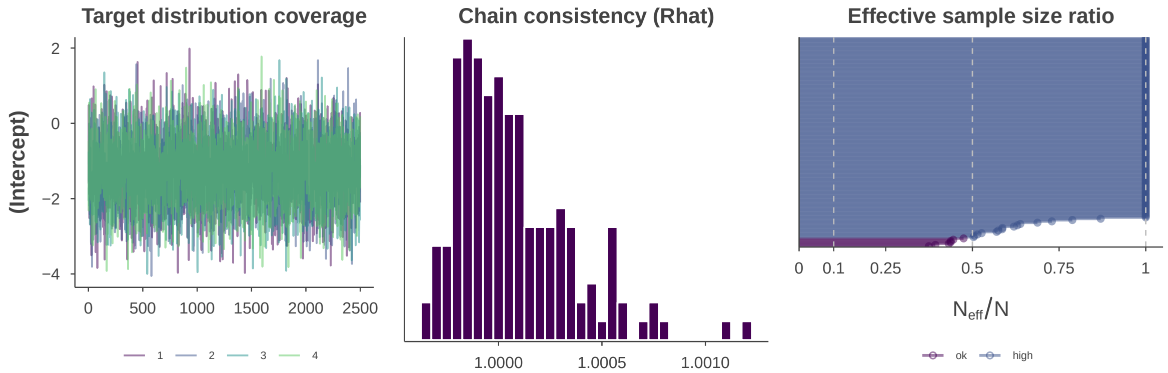

3.2.2 Model 2 convergence diagnostics plots

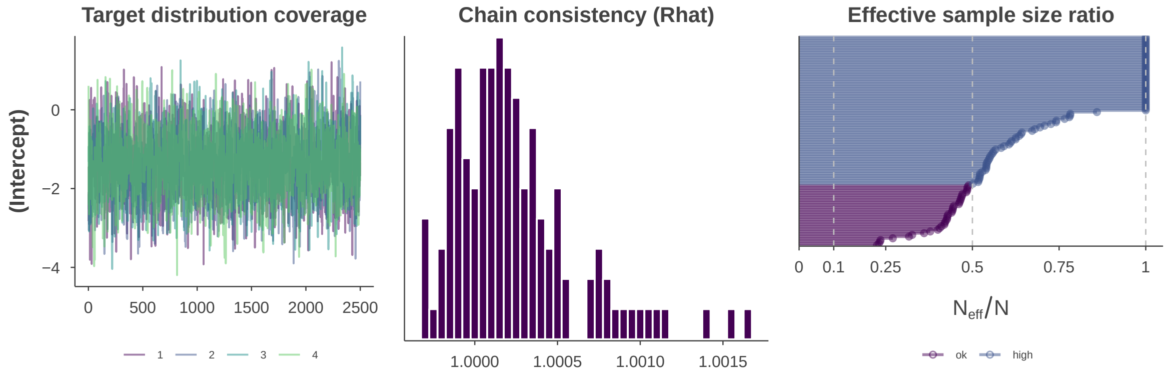

3.2.3 Model 3 convergence diagnostics plots

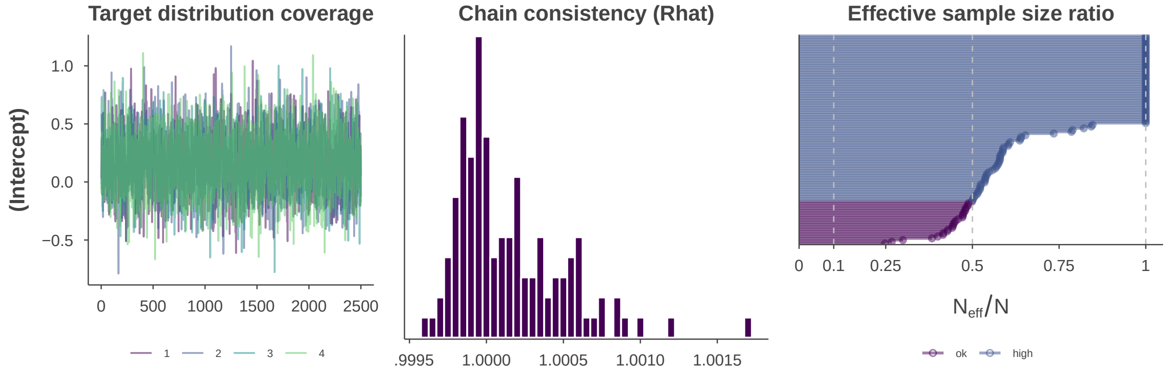

3.3 Posterior predictive density intervals

We draw samples from the posterior distributions of predicted values for each observation. The predicted value for each row in the original dataset will be a *distribution* of predicted values given one of the model equations, that is, each observation has a corresponding predictive distribution given the model formulation and the data. This results in N MCMC samples per data point (rows in the dataset), and $N_{rows} \times N_{mcmc}$ total predictions.

To load the fitted model data and posterior distributions from the `SRE` package, run the `sre_models` function with the following options.

```
sre_models(refit = FALSE, save_dir = NULL)
seed <- 19850519
```

This will load the model objects into the global workspace. The posterior predictive distributions and the associated data can be merged with the `merge_data_and_posterior` function.

```
ppd.full <- merge_data_and_posterior(stanreg_full,
                                     post_fun = rstanarm::posterior_linpred,
                                     transform = TRUE,
                                     seed = seed)

ppd.full.binary <- merge_data_and_posterior(stanreg_full, seed = seed)
```

3.3.1 Subjects

Table S11: Each participants' posteriror median accuracy, separated by AQ group intervals.

AQ	median	SD.lower	SD.upper	CI.lower	CI.upper
High	0.78206	0.73882	0.82204	0.71044	0.84806
High	0.70814	0.66557	0.7594	0.63285	0.78738
High	0.69897	0.65383	0.74441	0.62416	0.77447
High	0.69733	0.64781	0.74415	0.61518	0.77372
High	0.68149	0.63667	0.73235	0.60472	0.76191
High	0.63784	0.58579	0.68311	0.55818	0.72077
High	0.61187	0.55747	0.66084	0.52832	0.69646
High	0.56342	0.51159	0.61318	0.47908	0.64595
High	0.52443	0.47217	0.57666	0.44036	0.60913
High	0.50311	0.44635	0.55716	0.4133	0.59211
High	0.44248	0.39015	0.49653	0.35611	0.53019
High	0.40966	0.3537	0.46051	0.3192	0.4929
High	0.39605	0.34462	0.43982	0.32213	0.47705
High	0.37266	0.3205	0.42379	0.28927	0.45845
High	0.325	0.27554	0.37811	0.24438	0.41026

Table S12: Each participants' posteriror median accuracy, separated by AQ group intervals.

AQ	median	SD.lower	SD.upper	CI.lower	CI.upper
Low	0.85814	0.82263	0.89492	0.79956	0.91643
Low	0.85242	0.81636	0.89278	0.78379	0.90954
Low	0.84213	0.80231	0.88377	0.77636	0.90917
Low	0.74558	0.70364	0.79365	0.67094	0.81571
Low	0.71675	0.67009	0.76425	0.63939	0.79285
Low	0.70375	0.65848	0.7534	0.62381	0.7783
Low	0.68465	0.63613	0.72999	0.60558	0.76135
Low	0.65715	0.61155	0.70725	0.57744	0.73577
Low	0.64106	0.59122	0.69036	0.55604	0.71915
Low	0.63546	0.58775	0.68635	0.55758	0.71916
Low	0.63035	0.58303	0.68596	0.54516	0.71267
Low	0.62976	0.57814	0.68206	0.54276	0.71315
Low	0.62888	0.58038	0.68026	0.54347	0.70898
Low	0.61306	0.56053	0.66737	0.52634	0.69952
Low	0.60832	0.56173	0.6607	0.525	0.69289
Low	0.60328	0.54744	0.6519	0.51742	0.68993
Low	0.54016	0.48934	0.59578	0.45377	0.62556
Low	0.44871	0.3958	0.49902	0.36109	0.53135
Low	0.42334	0.36616	0.46998	0.33964	0.51214

3.3.2 AQ group

Posterior prediction comparisons between low vs. high scoring subjects.

Table S13: AQ group, posterior intervals.

aq_category	median	SD.lower	SD.upper	CI.lower	CI.upper
high symptoms	0.55676	0.54257	0.57042	0.53379	0.57974
low symptoms	0.65472	0.64346	0.66738	0.63541	0.67473
all	0.61253	0.53671	0.67373	0.53947	0.67096

3.3.3 Sex differences

Posterior prediction comparisons between male and female for each AQ group.

aq_category	median	SD.lower	SD.upper	CI.lower	CI.upper

Table S14: Male-Female acc. diff., posterior intervals.

aq_category	median	SD.lower	SD.upper	CI.lower	CI.upper
high symptoms	0.035684	0.0083043	0.065789	-0.012182	0.082466
low symptoms	0.078578	0.05504	0.10034	0.040189	0.11536

3.3.4 Contrasts

Posterior predictive density intervals for main, interaction, and simple effects.

Table S15: Posterior contrast intervals. (continued below)

contrast	effect	median	SD.lower	SD.upper	CI.lower	CI.upper	Bayes *p*		
Interaction contrast	action type x AQ	0.062	0.026	0.098	0.0021	0.12	0.041	p < .05	*
Simple contrasts	low AQ - high AQ	0.098	0.08	0.12	0.068	0.13	0	p < .001	***
Simple contrasts	complex actions - simple actions	0.19	0.17	0.2	0.15	0.22	0	p < .001	***
Simple effects	low AQ - high (complex actions)	0.067	0.045	0.093	0.029	0.11	0.003	p < .01	**
Simple effects	low AQ - high (simple actions)	0.13	0.1	0.16	0.084	0.17	0	p < .001	***
Simple effects	complex actions - simple (low AQ)	0.15	0.13	0.18	0.12	0.19	0	p < .001	***
Simple effects	complex actions - simple (high AQ)	0.22	0.19	0.24	0.17	0.26	0	p < .001	***
complex acc	complex high	0.66	0.64	0.68	0.63	0.7	0	p < .001	***
complex acc	complex low	0.73	0.72	0.75	0.71	0.76	0	p < .001	***
simple acc	simple high	0.45	0.43	0.47	0.41	0.48	0	p < .001	***
simple acc	simple low	0.58	0.56	0.6	0.55	0.61	0	p < .001	***

Table S17: Posterior contrast effect size intervals. (continued below)

Effect size	median	SD.lower	SD.upper	CI.lower	CI.upper
complex actions - simple (high AQ)	0.45	0.36	0.53	0.33	0.6
complex actions - simple (low AQ)	0.32	0.26	0.4	0.21	0.45
low AQ - high (complex actions)	0.14	0.066	0.21	0.017	0.26
low AQ - high (simple actions)	0.27	0.19	0.35	0.15	0.41
complex actions - simple actions	0.39	0.33	0.44	0.29	0.48
low AQ - high AQ	0.21	0.16	0.27	0.11	0.29
action type x AQ	0.13	0.024	0.24	-0.049	0.3

3.3.5 Response time

Table S19: Trials removed for no response after 40 seconds.

sub_sex	aq_category	nSub	total
m	low symptoms	4	4
f	low symptoms	2	4
f	high symptoms	1	1
m	high symptoms	1	1

Table S20: Posterior contrast intervals for response times. (continued below)

contrast	effect	median	SD.lower
Interaction contrast	action type x AQ	0.054	0.0026
Simple contrasts	low AQ - high AQ	0.1	0.075
Simple contrasts	complex actions - simple actions	0.19	0.16
Simple effects	low AQ - high (complex actions)	0.074	0.039

contrast	effect	median	SD.lower
Simple effects	low AQ - high (simple actions)	0.13	0.092
Simple effects	complex actions - simple (low AQ)	0.16	0.13
Simple effects	complex actions - simple (high AQ)	0.22	0.18
complex acc	complex high	0.67	0.64
complex acc	complex low	0.74	0.72
simple acc	simple high	0.45	0.42
simple acc	simple low	0.58	0.55

SD.upper	CI.lower	CI.upper	Bayes p		
0.1	-0.031	0.14	0.14	n.s.	
0.13	0.058	0.14	0	p < .001	***
0.21	0.15	0.23	0	p < .001	***
0.11	0.013	0.13	0.021	p < .05	*
0.16	0.072	0.19	3e-04	p < .001	***
0.2	0.11	0.22	0	p < .001	***
0.25	0.15	0.28	0	p < .001	***
0.69	0.62	0.71	0	p < .001	***
0.76	0.7	0.78	0	p < .001	***
0.48	0.41	0.49	0	p < .001	***
0.6	0.54	0.62	0	p < .001	***

Table S22: RT contrast effects in original scale (seconds)

contrast	effect		RT_effect
Interaction contrast	action type x AQ		5.2
Simple contrasts	low AQ - high AQ	***	5.5
Simple contrasts	complex actions - simple actions	***	6.1
Simple effects	low AQ - high (complex actions)	*	5.4
Simple effects	low AQ - high (simple actions)	***	5.7
Simple effects	complex actions - simple (low AQ)	***	5.9
Simple effects	complex actions - simple (high AQ)	***	6.3
complex acc	complex high	***	11
complex acc	complex low	***	12
simple acc	simple high	***	8.3
simple acc	simple low	***	9.7

3.3.6 Viewpoint

Difference in participants' rotations of actions from the baseline position (recorded angle).

Table S23: Viewpoint, posterior intervals.

Rotation	Median Diff.	SD.lower	SD.upper	CI.lower	CI.upper	$p(D = 0)$
0	0.00704	-0.00416	0.0158	-0.0087	0.026	0.225
135	-0.0024	-0.0122	0.00808	-0.0205	0.0154	0.404
247	-0.0155	-0.0266	-0.0012	-0.0375	0.00507	0.0753

3.4 `stanreg` model summaries

Coefficient information from each of the fitted models.

3.4.1 Model 1 - Full model

Number of coefficients for: *recog_acc ~ 1 + aq_category* action_category + aq_score_standard + sub_sex + sub_age_scaled + (1 + action_category | id) + (1 + angle | action)*

- `fixed` (*integer*): 7
- `random:id` (*integer*): 2
- `random:action` (*integer*): 4

```
============================================================================
M1 (Full) model:
============================================================================
stan_glmer
 family:       binomial [logit]
 formula:      recog_acc ~ 1 + aq_category * action_category + aq_score_standard +
       sub_sex + sub_age_scaled + (1 + action_category | id) + (1 +
       angle | action)
 observations: 2448
------
                                                     Median MAD_SD
(Intercept)                                          -1.275  0.819
aq_categorylow symptoms                               2.171  0.876
action_categorysimple actions                        -1.088  0.278
aq_score_standard                                     1.351  0.571
sub_sexm                                              0.309  0.251
sub_age_scaled                                        0.442  0.254
aq_categorylow symptoms:action_categorysimple actions  0.297  0.296

Error terms:
 Groups Name                          Std.Dev. Corr
 id     (Intercept)                   0.7622
        action_categorysimple actions 0.7038   -0.56
```

```
 action (Intercept)                     0.3764
        angle0                          0.2475    0.12
        angle135                        0.2460   -0.15 -0.02
        angle247                        0.2734   -0.24 -0.09  0.18
Num. levels: id 34, action 18

Sample avg. posterior predictive distribution of y:
         Median MAD_SD
mean_PPD 0.612  0.013

------
* For help interpreting the printed output see ?print.stanreg
* For info on the priors used see ?prior_summary.stanreg

Priors for model 'stanreg'
------
Intercept (after predictors centered)
 ~ student_t(df = 4, location = 0, scale = 10)

Coefficients
 ~ student_t(df = [4,4,4,...], location = [0,0,0,...], scale = [2.5,2.5,2.5,...])
     **adjusted scale = [2.500,2.500,3.328,...]

Covariance
 ~ decov(reg. = 2, conc. = 2, shape = 1, scale = 1)
------
See help('prior_summary.stanreg') for more details
```

3.4.1.1 LOO-CV for Model 1

```
#>
#> Computed from 10000 by 2448 log-likelihood matrix
#>
#>           Estimate     SE
#> elpd_loo -1464.015 20.036
#> p_loo       74.792  1.468
#> looic     2928.029 40.071
#> ------
#> Monte Carlo SE of elpd_loo is 0.093.
#>
#> All Pareto k estimates are good (k < 0.5).
#> See help('pareto-k-diagnostic') for details.
```

3.4.2 Model 2 - Alternative model with no interaction

Number of coefficients for: *recog_acc ~ 1 + aq_category + action_category + aq_score_standard + sub_sex + sub_age_scaled + (1 | id) + (1 + angle | action)*

- `fixed` (*integer*): 6
- `random:id` (*integer*): 1
- `random:action` (*integer*): 4

```
============================================================================
M2 (Alternative) model:
============================================================================
stan_glmer
 family:       binomial [logit]
 formula:      recog_acc ~ 1 + aq_category + action_category + aq_score_standard +
       sub_sex + sub_age_scaled + (1 | id) + (1 + angle | action)
 observations: 2448
------
                              Median MAD_SD
(Intercept)                   -1.348  0.774
aq_categorylow symptoms        2.290  0.811
action_categorysimple actions -0.889  0.182
aq_score_standard              1.323  0.539
sub_sexm                       0.294  0.243
sub_age_scaled                 0.425  0.237

Error terms:
 Groups Name        Std.Dev. Corr
 id     (Intercept) 0.5940
 action (Intercept) 0.3627
        angle0      0.2430    0.13
        angle135    0.2391   -0.14 -0.01
        angle247    0.2631   -0.24 -0.09  0.17
Num. levels: id 34, action 18

Sample avg. posterior predictive distribution of y:
         Median MAD_SD
mean_PPD 0.612  0.013

------
* For help interpreting the printed output see ?print.stanreg
* For info on the priors used see ?prior_summary.stanreg

Priors for model 'stanreg'
```

```
------
Intercept (after predictors centered)
 ~ student_t(df = 4, location = 0, scale = 10)

Coefficients
 ~ student_t(df = [4,4,4,...], location = [0,0,0,...], scale = [2.5,2.5,2.5,...])
     **adjusted scale = [2.500,2.500,3.328,...]

Covariance
 ~ decov(reg. = 2, conc. = 2, shape = 1, scale = 1)
------
See help('prior_summary.stanreg') for more details
```

3.4.2.1 LOO-CV for Model 2

```
#>
#> Computed from 10000 by 2448 log-likelihood matrix
#>
#>          Estimate    SE
#> elpd_loo -1479.957 19.651
#> p_loo       53.825  0.987
#> looic     2959.914 39.302
#> ------
#> Monte Carlo SE of elpd_loo is 0.077.
#>
#> All Pareto k estimates are good (k < 0.5).
#> See help('pareto-k-diagnostic') for details.
```

3.4.3 Model 3 - Null model has no AQ or action type effects.

Number of coefficients for: *recog_acc ~ 1 + sub_sex + sub_age_scaled + (1 | id) + (1 + angle | action)*

- `fixed` (*integer*): 3
- `random:id` (*integer*): 1
- `random:action` (*integer*): 4

```
============================================================================
M3 (Null) model:
============================================================================
stan_glmer
 family:       binomial [logit]
 formula:      recog_acc ~ 1 + sub_sex + sub_age_scaled + (1 | id) + (1 + angle |
       action)
```

```
 observations: 2448
------
               Median MAD_SD
(Intercept)    0.172  0.236
sub_sexm       0.304  0.260
sub_age_scaled 0.430  0.259

Error terms:
 Groups Name        Std.Dev. Corr
 id     (Intercept) 0.6773
 action (Intercept) 0.5771
        angle0      0.2677   -0.07
        angle135    0.2962   -0.06  0.00
        angle247    0.3344   -0.31 -0.08  0.25
Num. levels: id 34, action 18

Sample avg. posterior predictive distribution of y:
         Median MAD_SD
mean_PPD 0.612  0.013

------
* For help interpreting the printed output see ?print.stanreg
* For info on the priors used see ?prior_summary.stanreg

Priors for model 'stanreg'
------
Intercept (after predictors centered)
 ~ student_t(df = 4, location = 0, scale = 10)

Coefficients
 ~ student_t(df = [4,4], location = [0,0], scale = [2.5,2.5])
     **adjusted scale = [2.500,5.000]

Covariance
 ~ decov(reg. = 2, conc. = 2, shape = 1, scale = 1)
------
See help('prior_summary.stanreg') for more details
```

3.4.3.1 LOO-CV for Model 3

```
#>
#> Computed from 10000 by 2448 log-likelihood matrix
```

```
#>
#>           Estimate     SE
#> elpd_loo -1481.619 19.492
#> p_loo       59.718  1.077
#> looic     2963.238 38.983
#> ------
#> Monte Carlo SE of elpd_loo is 0.082.
#>
#> All Pareto k estimates are good (k < 0.5).
#> See help('pareto-k-diagnostic') for details.
```

3.5 Model comparisons

3.5.1 Expected log pointwise predictive densities

Approximate (Pareto-smoothed sampling) Leave-One-Out Cross-validation comparisons between Models 1–3.

Table S24: PSIS-LOO-CV model comparisons

	Ful(M1)* Alt(M2)	Ful(M1)* Nul(M3)	Alt(M2) Nul(M3)
ELPD_LOO L	-1464	-1464	-1480
ELPD_LOO R	-1480	-1482	-1482
ELPD_LOO DIFF.	-15.94	-17.6	-1.662
SE DIFF.	5.614	5.965	2.103
CI 2.5 %	-26.95	-29.3	-5.785
CI 97.5 %	-4.933	-5.907	2.461

3.5.2 Bayesian R-squared comparisons

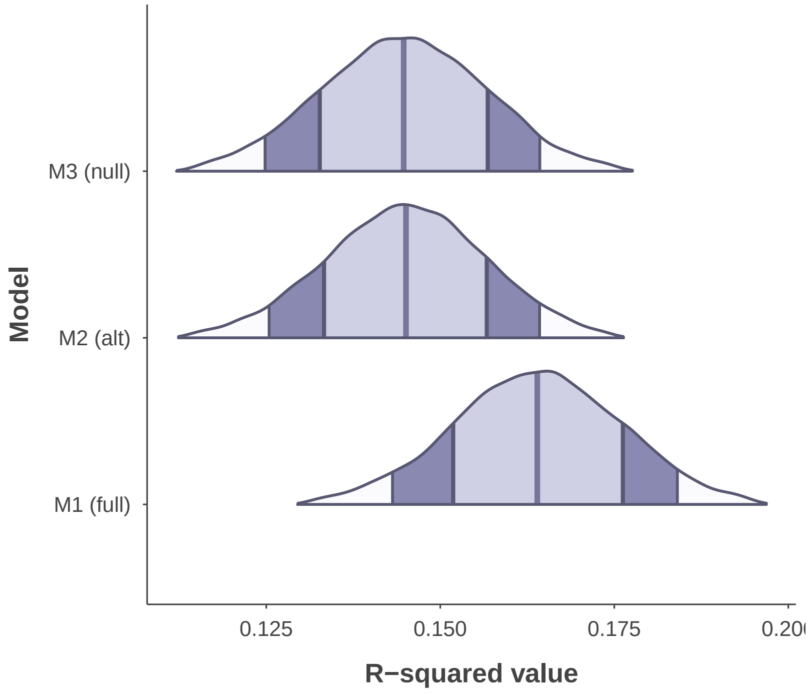

3.5.3 Posterior predictive checks

Comparisons between the mean accuracy for each subject and the mean posterior predictive accuracy density for each MCMC sample and subject. The plots are split by AQ group.

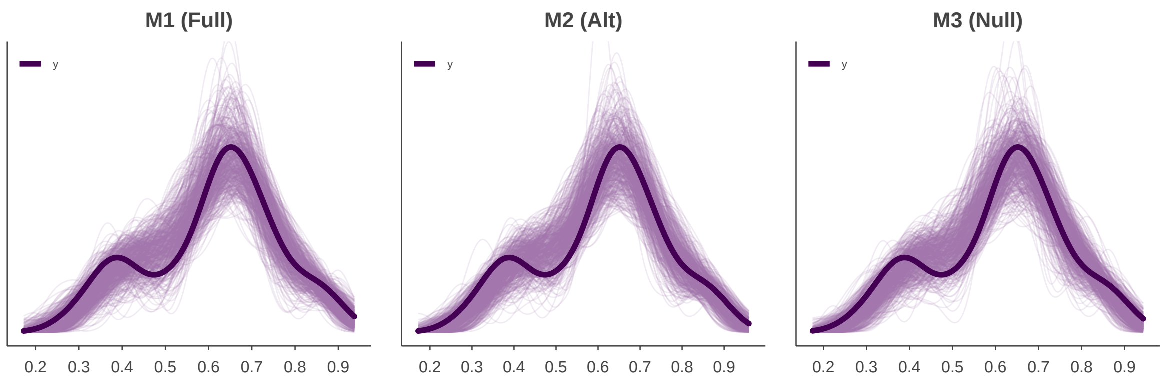

4 ADDITIONAL FIGURES

4.1 AQ group by action type, bar plot

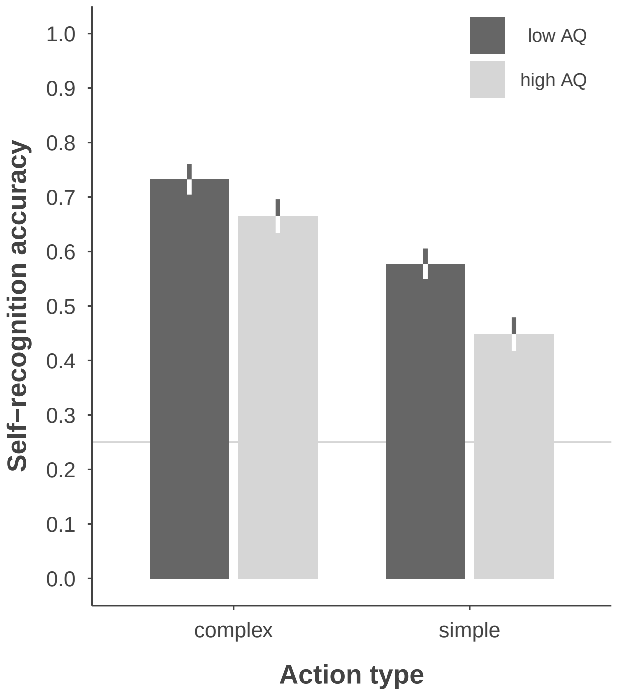

Figure S1. Mean self-recognition accuracy by AQ group and action type. Accuracy is averaged over viewpoints. Chance performance is shown by the grey horizontal line at 0.25, and within-subjects standard errors are shown for each bar

4.2 AQ group by action type, histograms

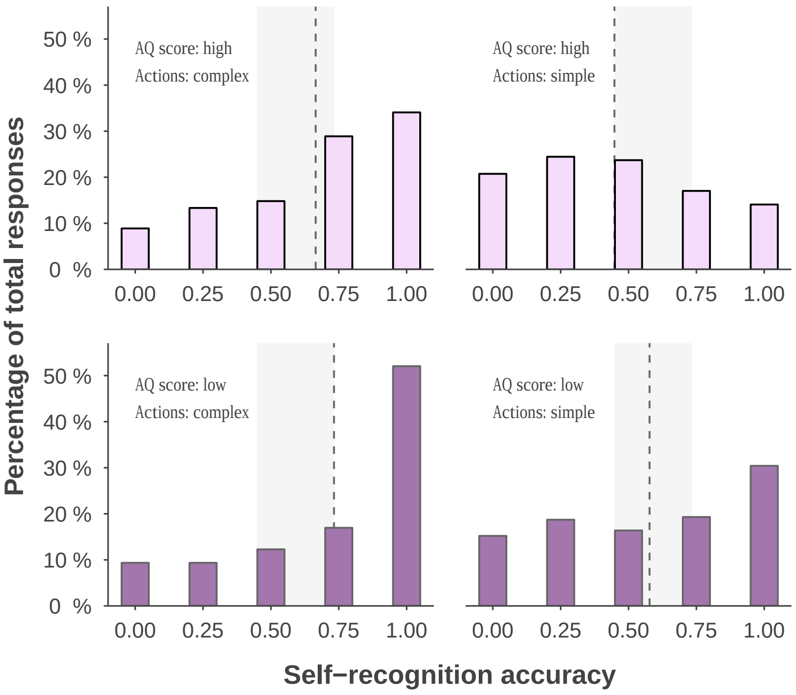

Figure S2. Histograms of each accuracy score by AQ group and action type. Accuracy is averaged over viewpoints. The dotted vertical lines indicate the mean accuracy for a given panel. The shaded region is the mean accuracy range across panels.

4.3 Posterior accuracy by actions

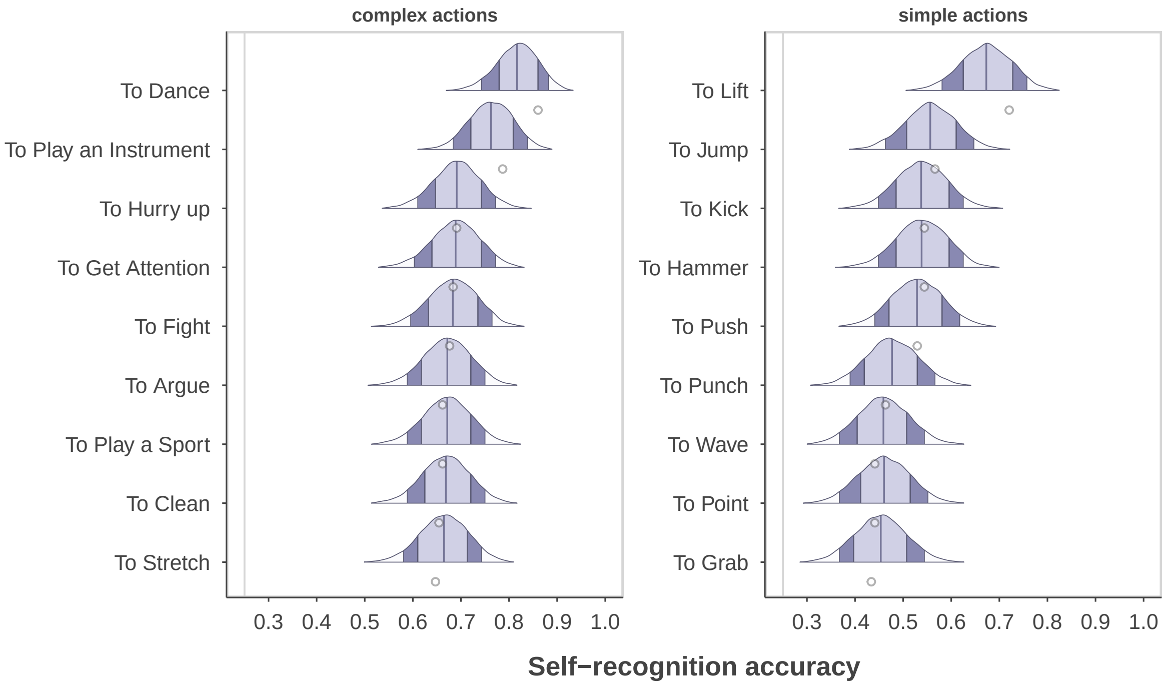

Figure S3. Posterior distributions of self-recognition accuracy for each action performed by the participants, categorized by action type. The white point in each distribution is the mean accuracy computed from the data, collapsed across AQ groups and viewpoint. The vertical grey line at 0.25 represents chance performance.

4.4 AQ score by action type with gender points

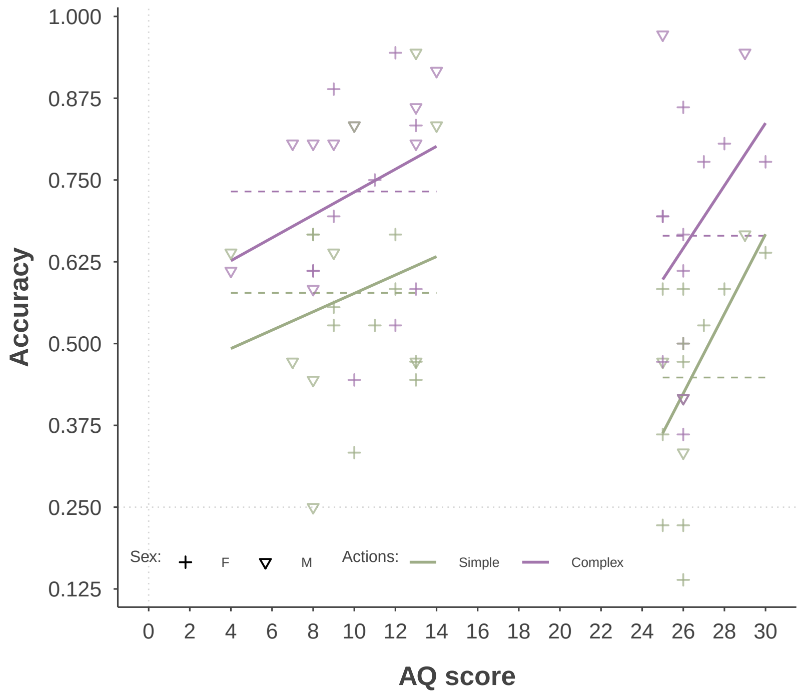

Figure S4. Interactions between AQ score and action complexity.

5 USING THE EXPORTED SRE PACKAGE FUNCTIONS

Loading the package materials

```
library(SRE)
```

Calling the package dataset

```
SRE
```

Alternatively, you may import using the `import_sre()` function to customize how the data is imported. If then this will import the dataset without refactoring the variables or standardizing.

```
SRE <- import_sre(raw=TRUE)
```

5.1 Refitting the models

To load or refit all the models, use the `sre_models` function. If `save_dir=NULL` and `refit=TRUE`, this will refit the default models and save all refitted models to a directory

called `sre_fitted_models` in your OS specific user/home directory. If `refit=FALSE` then it will load the saved models from the package's external data folder.

```
sre_models(refit = TRUE, save_dir = NULL)
```

If you specify a path for `save_dir`, all models can be saved and loaded from there. The `debug` option just runs a few iterations for each model.

```
model_refit_dir <- "./sre_models_example"
sre_models(refit = TRUE, save_dir = model_refit_dir, debug = FALSE)
```

5.1.1 Loading fitted models

```
sre_models(refit = FALSE, save_dir = NULL)
```

This will load the following R objects into the global environment.

- `stanreg_full`: Model 1
- `loo_full`: LOO for Model 1
- `stanreg_alt`: Model 2
- `loo_alt`: LOO for Model 2
- `stanreg_null`: Model 3
- `loo_null`: LOO for Model 3

REFERENCES

Carpenter, B., Gelman, A., Hoffman, M., Lee, D., Goodrich, B., Betancourt, M., … Riddell, A. (2017). Stan: A probabilistic programming language. *Journal of Statistical Software, Articles*, *76*(1), 1–32. https://doi.org/10.18637/jss.v076.i01

Ghosh, J., Li, Y., & Mitra, R. (2015). On the Use of Cauchy Prior Distributions for Bayesian Logistic Regression. *ArXiv E-Prints*. Retrieved from http://arxiv.org/abs/1507.07170

Homan, M. D., & Gelman, A. (2014). The no-u-turn sampler: Adaptively setting path lengths in hamiltonian monte carlo. *Journal of Machine Learning Research*, *15*(1), 1593–1623. Retrieved from http://dl.acm.org/citation.cfm?id=2627435.2638586

Lewandowski, D., Kurowicka, D., & Joe, H. (2009). Generating random correlation matrices based on vines and extended onion method. *Journal of Multivariate Analysis*, *100*(9), 1989–2001. https://doi.org/doi.org/10.1016/j.jmva.2009.04.008
